# Supplementary material for: Irritant and repellent behaviors of sterile male Aedes aegypti (L.) (Diptera: Culicidae) mosquitoes are crucial in the development of disease control strategies applying sterile insect technique
Source: PeerJ. 2024 Mar 22;12:e17038. doi: 10.7717/peerj.17038 (PMC10962334; doi:10.7717/peerj.17038)
Supplement: Table S1 [file peerj-12-17038-s005.docx]

**Supplementary Table 1.** Mean percentage escape of radiation-sterilized male *Ae. aegypti* exposed to citronella, DEET, transfluthrin and deltamethrin in ER system.

| Assay | Compound | Percentage escape response | | | |
| --- | --- | --- | --- | --- | --- |
|  |  | SIT | | Non-SIT | |
|  |  | Treatment±SE (a) | Control | Treatment±SE(a) | Control |
| Noncontact | Citronella 2.5 % | 23.33±6.38 (17.85) | 6.67 | 45.00±10.67 (37.73) | 11.67 |
|  | Citronella 5 % | 61.67± 14.24 (56.61) | 11.67 | 70.00±13.47 (66.04) | 11.67 |
|  | DEET 2.5% | 11.67±4.19 (5.36) | 6.67 | 8.33±3.19 (3.51) | 5 |
|  | DEET 5.0% | 13.33±4.17 (3.70) | 10 | 11.67±5.69 (3.64) | 8.33 |
|  | Transfluthrin | 40.00±18.05 (33.45) | 9.84 | 48.33±5 (41.98) | 10.94 |
|  | Deltamethrin | 31.67±12.58 (25.46) | 8.33 | 28.33±3.19 (21.82) | 8.33 |
| Contact | Citronella 2.5 % | 65±9.17 (61.11) | 10 | 63.33±10 (59.67) | 10 |
|  | Citronella 5 % | 73.33±9.81 (70.37) | 10 | 70.00±6.38 (66.67) | 10 |
|  | DEET 2.5% | 26.67±8.16 (21.52) | 6.56 | 29.03±7.93 (25.36) | 4.92 |
|  | DEET 5.0% | 28.33±7.39 (20.37) | 10 | 31.67±7.93 (26.79) | 6.67 |
|  | Transfluthrin | 45.00±9.57 (42.11) | 5 | 28.33±9.57 (18.66) | 11.67 |
|  | Deltamethrin | 60.00±13.6 (58.62) | 3.33 | 53.33±13.6 (49.99) | 6.67 |

(a), Treatment escape percent adjusted using Abbott formular.

Error bars represent the SE.
